# The Malabar Coolies

**Published:** 1855-01

**Authors:** 


*The Malabar Coolies.*—Blood drawn from their veins will be found greatly deficient in fibrin, albumen, and coloring matter. Their nervous system does not betray that amount of sensibility to external impressions which is observed amongst other classes, and it is a remarkable fact, that a Tamalian will bear any amount of what is known as "shock to the system," without any detriment; while the slightest drain from the body—as a few fluid evacuations from the bowels, or rather copious discharge from an abscess—is sure to be attended with the most serious consequences. The whole limb may be severed in surgical operations, and scarcely a groan will escape him. A patient cannot manifest less sensibility to pain under the knife of the surgeon than a poor Malabar, when previously desired to "screw himself up to the sticking-point;" but the Tamal coolies, from a natural state of physical infirmity, are perfectly incapable of supporting anything like an exhausting influence. —Mr. DICKMAN *in Ceylon Miscellany.*

